# A Fast and Validated HPLC Method for the Simultaneous Analysis of Five 5-HT_3_ Receptor Antagonists via the Quantitative Analysis of Multicomponents by a Single Marker

**DOI:** 10.1155/2021/5533646

**Published:** 2021-06-26

**Authors:** Fuchao Chen, Baoxia Fang, Peng Li, Sicen Wang

**Affiliations:** ^1^Sinopharm Dongfeng General Hospital, Hubei University of Medicine, Shiyan, Hubei 442008, China; ^2^School of Pharmacy, Xi'an Jiaotong University, Xi'an 710061, Shanxi, China

## Abstract

In this study, a new strategy for the simultaneous quantization of five serotonin 5-hydroxytryptamine receptor antagonists—ondansetron, azasetron, ramosetron, granisetron, and tropisetron—either in infusion samples or in injection dosage form was first established based on high-performance liquid chromatography combined with a quantitative analysis of multiple components by a single marker. The quantitative analysis of multicomponents by a single marker method was conducted with ondansetron as an internal reference substance and performed using relative retention time and ultraviolet spectral similarity as the double indicator. The quantitative analysis of the 5-HT_3_ receptor antagonists was calculated and investigated based on the relative correction factors. Chromatographic separation was achieved using a C_18_ column (150 mm × 4.6 mm, 5.0 *μ*m), and the mobile phase was composed of acetonitrile-0.05 mol·L^−1^ potassium dihydrogen phosphate (pH 4.0) (25 : 75) at a flow rate of 1.0 mL·min^−1^ and detection wavelengths of 307 nm (ondansetron, azasetron, ramosetron), 302 nm (granisetron), and 285 nm (tropisetron). In addition, the accuracy of the quantitative analysis of multicomponents by a single marker method was compared with an external standard method, and no significant difference was observed between the two methods. The established method is rapid, is easy, and does not require many reference substances, and it can been successfully applied as part of the quality control of the five 5-HT_3_ receptor antagonists in their injection dosage form and infusion sample drugs in hospitals.

## 1. Introduction

**Figure 1:**
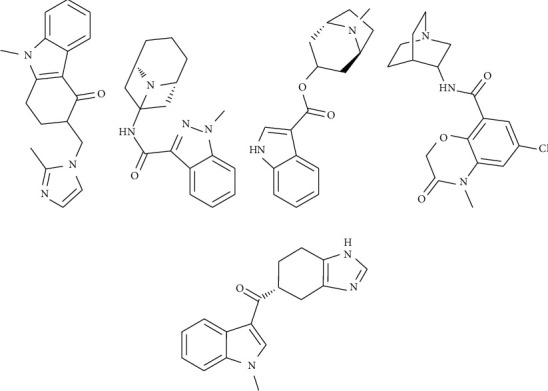
Chemical structure of ODT (a), GNT (b), TPT (c), AZT (d), and RMT (e).

The effects of chemotherapy, radiation, irritable bowel syndrome, opioid analgesic drugs, anesthesia, and postoperative-induced nausea and vomiting are among the most distressful side effects in patients. These side effects of nausea and vomiting can significantly cause poor appetite, bodyweight loss, decreased social skills, and more severe clinical consequences such as dehydration, electrolyte imbalances, and prolonged hospital stays [1]. The 5-hydroxytryptamine (5-HT_3_) receptor antagonists, informally known as “setrons,” are a class of antiemetic medications that inhibit the release of 5-HT and vagal 5-HT receptors in the central nervous system. Serotonin 5-HT_3_ receptor antagonists, including ondansetron (ODT), granisetron (GNT), tropisetron (TPT), azasetron (AZT), and ramosetron (RMT), are particularly effective in the treatment and prevention of nausea and vomiting [2–6]. The chemical structures of the five 5-HT_3_ receptor antagonists are shown in Figure 1.

At present, the rapid detection of drugs mainly involves infrared spectroscopy. However, near-infrared spectroscopy technology is only applicable to the initial screening of drugs, and a quantitative model is more complex and rare, resulting in the quantitative requirements of rapid drug detection not being met. HPLC is commonly used to verify rapid detection systems due to its attractive features, such as high peak efficiencies, great resolution, high sensitivity, good repeatability, and wide application range. HPLC analytic methods for the detection of 5-HT_3_ receptor antagonists such as ODT, GNT, TPT, AZT, and RMT are already mature, and most of them use retention data in the qualitative analysis, while the external standard method (ESM) is applicable in quantitative analysis [7–24]. However, some of these methods may take a long time, and the specific drugs to be tested must correspond to reference substances, which cannot meet the requirements of rapid drug detection.

To solve the above problems, a unique quantitative analysis of multicomponents by a single marker (QAMS) analytical method was adopted in this paper. The QAMS method is able to simultaneously identify and quantify itself and the other analytes by a single reference standard, which greatly reduces the cost and analysis time of the experiment [25–32]. To date, there are no reports concentrating the QAMS or ESM method on the simultaneous quantification of ODT, GNT, TPT, AZT, and RMT in infusion samples and in injection dosage form. Thus, the purpose of this study was to establish a rapid HPLC-QAMS method for the detection of five 5-HT_3_ receptor antagonists by using two indexes of relative retention time and UV spectrum similarity for qualitative analysis and a relative correction factor for quantitative analysis. This method was successfully employed for the routine quality control of 5-HT_3_ receptor antagonist injection, infusion products, and the preliminary screening of unknown drugs in hospitals.

## 2. Experimental Procedure

### 2.1. Chemicals and Reagents

Six reference substances (ODT, GNT, TPT, AZT, RMT, and urine pyrimidine) were purchased from the National Institute for the Control of Pharmaceutical and Biological Products (Beijing, China). Each of six reference substances had a purity of more than 99.5%. HPLC-grade acetonitrile and AR-grade potassium dihydrogen phosphate, phosphoric acid, and triethylamine were supplied by the Sinopharm Chemical Reagent Co., Ltd. (Beijing, China). Ultrapure water was obtained from a Millipore Milli-Q system (Millipore, USA).

### 2.2. Pharmaceutical Formulations

The following dosage forms were analyzed: commercial injection of ODT hydrochloride from Qilu Pharmaceutical Ltd. (Shandong, China) claimed to contain 2 mg of ONT per mL. Commercial injection of GNT hydrochloride from Cinkate Pharmaceutical (Suzhou, China) claimed to contain 1 mg of GNT per mL. Commercial injection of TPT hydrochloride from Qilu Pharmaceutical (Shandong, China) claimed to contain 5 mg of TPT per mL. Commercial injection of AZT hydrochloride from Wanma Pharmaceutical (Zhejiang, China) was labeled to contain 5 mg of AZT per mL. Commercial injection of RMT hydrochloride from Cisen Pharmaceutical (Shandong, China) was labeled to contain 0.3 mg of RMT per mL.

### 2.3. Instrumentation and Chromatographic Conditions

Chromatographic analysis was performed on an HPLC system (Dionex, Germany) consisting of an UltiMate® 3000 quaternary pump, an autosampler, a column-heating compartment, and an ultraviolet detector (DAD). Chromatographic data were collected and analyzed using Chromeleon® 7.2 software. The simultaneous separation of five 5-HT_3_ receptor antagonists was performed using an InertSustain C_18_ column (4.6 mm × 150 mm, 5 *μ*m) supplied by SHIMADZU (China) Co., Ltd. The mobile phase consisted of acetonitrile-0.05 mol·L^−1^ potassium dihydrogen phosphate (0.1% phosphoric acid, pH 4.0) (25 : 75, v/v) with a flow rate of 1.0 mL·min^−1^ for 15 min. All determinations were carried out at ambient temperature 30°C. The detector wavelengths for ODT, GNT, TPT, AZT, and RMT were 307, 302, 285, 307, and 307 nm, respectively.

### 2.4. Preparation of Standard Solutions

Stock solutions were prepared by weighing accurately and dissolving the five 5-HT_3_ receptor antagonists' standard references at 5.0 mg each and immersing them in ultrapure water in a 10 mL volumetric flask with a concentration of approximately 0.5 mg/mL as the reference standard. A standard stock solution of urine pyrimidine (20 *μ*g/mL) was prepared by dissolving 2.0 mg of urine pyrimidine with ultrapure water in a 100 mL volumetric flask. Mixed 5-HT_3_ receptor antagonist working standard solutions were prepared by appropriate dilution of the stock solutions with ultrapure water to the required concentrations for plotting the calibration curves. All standard solutions were stored at −20°C until use and sonicated for 10 min for injection.

### 2.5. Method Validation

The method was validated according to the International Conference of Harmonization (ICH) guidelines [33]. The following parameters were investigated: linearity, precision, stability, accuracy, limit of detection, and robustness. The limit of detection for each 5-HT_3_ receptor antagonist was determined at a signal-to-noise ratio (S/N) of 3. For the limit of quantification, the ratio considered was 10 : 1 with an RSD% value less than 10% [34].

#### 2.5.1. Construction of Calibration Graphs

The calibration graphs of the method were evaluated with a series of different concentrations of working standards (mixture of all five 5-HT_3_ receptor antagonists). The concentration range was selected at six different concentrations, viz 5, 10, 20, 50, 75, and 100 *μ*g/mL for ODT, GNT, TPT, and AZT and 0.5, 3, 6, 20, 30, and 50 *μ*g/mL for RMT. A 20 *μ*L aliquot of each working solution was injected in triplicate into a chromatographic system (*n* = 3). The peak areas of the five 5-HT_3_ receptor antagonists were plotted against the corresponding concentrations of each drug to obtain the calibration curve.

#### 2.5.2. Precision

For method precision, three concentration levels of mixed standard solutions (10.0, 20.0, and 50.0 *μ*g/mL for ODT, GNT, TPT, AZT; 1.0, 3.0, and 6.0 *μ*g/mL for RMT) were assessed in triplicate during a single day and three consecutive days. The percent relative standard deviation (% RSD) of five analytes was calculated according to the peak area of each component in a single day and on different days to obtain the intraday precision and interday precision.

#### 2.5.3. Stability

The sample solutions of the five 5-HT_3_ receptor antagonists were prepared with ultrapure water stored at room temperature and injected into HPLC at 0, 1, 2, 4, 6, and 8 h after being prepared. The stability of the sample solutions was investigated by the RSD of variation in the peak area of the five analytes.

#### 2.5.4. Accuracy

The accuracy of the method was determined as the recoveries of known added amounts of five 5-HT_3_ receptor antagonist reference substance into the previously analyzed commercial injections in triplicate using three concentration levels.

#### 2.5.5. Robustness Test

The robustness test was conducted by four deliberate variations to some chromatographic parameters such as the column temperature, acetonitrile percent, flow rate, and pH value of the mobile phase. The acetonitrile percent, flow rate, and pH value of the mobile phase were changed by ±2, ±0.02, and ±0.2, respectively. The column temperature was altered to ±1°C (from 29 to 31°C). The separation degree and RSD% of the five 5-HT_3_ receptor antagonists were investigated.

### 2.6. Qualitative Investigation

#### 2.6.1. UV Spectral Similarity

The mixed working standard solutions of the five 5-HT_3_ receptor antagonists were injected into the HPLC system according to the chromatographic conditions given in Section 2.3. Then, the spectral and 1st UV spectra were recorded. The cosine of the vector angle was calculated for similarity evaluation of UV spectra among the five 5-HT_3_ receptor antagonists. The angle cosine formula is expressed in equation (1) in which *X*_*ki*_ and *X*_*kj*_ are the peak point absorbances of the reference and test samples, respectively. The similarity values can quantitatively reflect the similarity degree of the different UV spectra. When the similarity value is close to 1, the similarity degree of the different UV spectra is high. In addition, when the similarity value is close to 0, the difference between the UV spectra is large.

(1) $$\begin{array}{c} C_{ij}=\frac{\sum_{k=1}^{n}X_{ki}X_{kj}}{\sqrt{\sum_{k=1}^{n}X_{ki}^{2}\sum_{k}^{n}X_{kj}^{2}}}. \end{array}$$

#### 2.6.2. Relative Retention Time

The same mixed standard solutions of the five analytes and urine pyrimidine were tested under the chromatographic conditions as described in Section 2.3 to identify the relative retention time (RRT) and the RSD%. The RRTs are calculated by the following equation:

(2) $$\begin{array}{c} \text{RRT}=\frac{t_{x}-t_{0}}{t_{i}-t_{0}}, \end{array}$$

where *t*_0_, *t*_*i*_, and *t*_*x*_ represent the retention time of urine pyrimidine, ONT, and analyte, respectively.

### 2.7. Calculation of Relative Correction Factors Using the QAMS Method

In this study, due to the low price, availability, chemical stability, good separation, and chromatographic peaks having no interference with other 5-HT_3_ receptor antagonists, ODT was chosen as the internal standard substance for the QAMS method. The relative correction factors (RCF, f) between ODT and the other 5-HT_3_ receptor antagonists were calculated using equation (3). The concentration of each 5-HT_3_ receptor antagonist in the sample solution can be calculated using equation (4) [25–32].

(3) $$\begin{array}{c} {\text{RCF}}_{x}=\frac{f_{x}}{f_{i}}=\frac{A_{x}/C_{x}}{A_{i}/C_{i}}, \end{array}$$

(4) $$\begin{array}{c} C_{x}=\frac{C_{i}}{{\text{RCF}}_{x}}\times \frac{A_{x}}{A_{i}}, \end{array}$$

where *A*_*i*_ is the peak area of an internal reference substance (ODT) under test, and *C*_*i*_ is the concentration of internal reference substance under test. *A*_*x*_ is the peak area of other investigated components, and *C*_*x*_ is the concentration of other investigated components in the sample solution.

### 2.8. Sample Analysis

#### 2.8.1. Analysis of the Five 5-HT_3_ Receptor Antagonists in Infusion Samples

All infusion samples of the five 5-HT_3_ receptor antagonists were prepared under aseptic conditions in laminar flow hoods by licensed central intravenous additive services in hospitals. The most commonly submitted infusion preparations for ODT, GNT, TPT, AZT, and RMT were 80.0, 30.0, 50.0, 100, and 3.0 *μ*g/mL diluted in 0.9% sodium chloride injection. Accurate volumes of each 5-HT_3_ receptor antagonist's infusion preparations were transferred into a set of 10 mL volumetric flasks and then diluted to volume with ultrapure water to keep the concentrations of the drugs within the linear ranges.

#### 2.8.2. Analysis of the Five 5-HT_3_ Receptor Antagonists in Commercial Injections

Commercially available injections of the five 5-HT_3_ receptor antagonists were prepared with ultrapure water and injected into HPLC under the chromatographed conditions described above. Subsequently, the chromatographic peak area of each of the five 5-HT_3_ receptor antagonists was recorded. Then, the content of the five 5-HT_3_ receptor antagonists was calculated by the ESM and QAMS methods.

## 3. Results and Discussion

### 3.1. Optimization of Chromatographic Conditions

**Figure 2:**
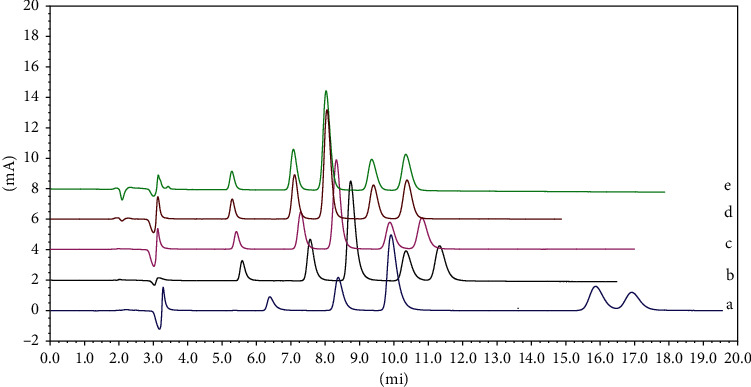
Typical HPLC chromatogram for simultaneous separation of ODT, GNT, TPT, AZT, and RMT. Chromatographic conditions were acetonitrile-0.05 mol·L^−1^ potassium dihydrogen phosphate (25 : 75, v/v) except the buffer pH was varied: (a) pH 3.0; (b) pH 3.5; (c) pH 4.0; (d) pH 4.5; and (e) pH 5.0.

**Figure 3:**
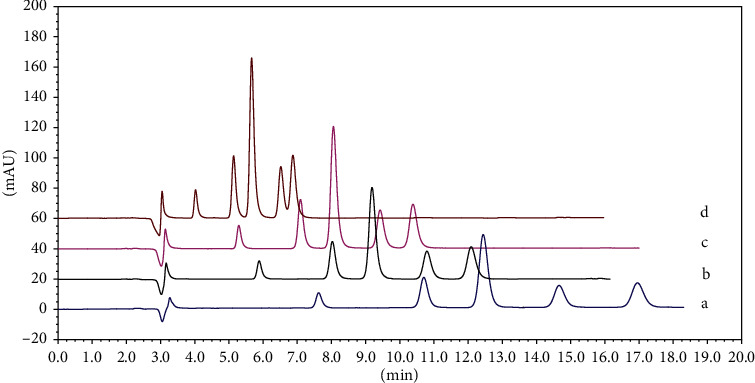
Typical HPLC chromatogram for simultaneous separation of ODT, GNT, TPT, AZT, and RMT. Chromatographic conditions were acetonitrile-0.05 mol·L^−1^ potassium dihydrogen phosphate (phosphoric acid to adjust pH to 4.0), except the acetonitrile content was varied: (a) acetonitrile content 20; (b) acetonitrile content 25; (c) acetonitrile content 30; and (d) acetonitrile content 35.

According to the literature [7–24], acetonitrile and acidic water media were selected to optimize the mobile phase composition. A series of concentrations, pH values, the ratio of aqueous potassium dihydrogen phosphate solution, and different chromatographic column types were studied to ensure good resolution and appropriate retention time of the five 5-HT_3_ receptor antagonists. The HPLC chromatograms are shown in Figures 2 and 3. The best result was achieved by comparing the peak shapes and resolutions of the investigated drugs at a pH of 4.0 and acetonitrile-50 mM KH_2_PO_4_ buffer (25 : 75; v/v) at a flow rate of 1.0 mL/min.

### 3.2. Calibration Curves, Precision, Stability, and Accuracy

The linearity range, precision, stability, and recovery are shown in Tables 1 and 2. High coefficient of determination values (*r*^2^ > 0.999) showed good linearity for all five 5-HT_3_ receptor antagonists. The RSDs of intraday and interday variability ranged from 0.4 to 1.9%, which showed good instrument precision. The RSDs of the peak areas of ODT, GNT, TPT, AZT, and RMT were 0.8%, 0.6%, 0.9%, 1.8%, and 1.2% (RSD% ≤ 2.0%), which indicates that the five 5-HT_3_ receptor antagonists tested sample solution were stable within 8 hours (average time of analysis) and can be evaluated under normal laboratory environment without any significant loss. The recoveries ranged from 98.3% to 101.8%, with RSD < 2.0%, illustrating that the method was accurate.

### 3.3. UV Spectral Similarity and Relative Retention Time

**Figure 4:**
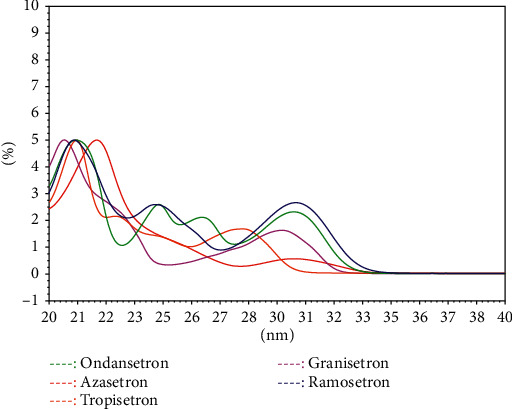
The UV spectra of ODT, GNT, TPT, AZT, and RMT.

**Figure 5:**
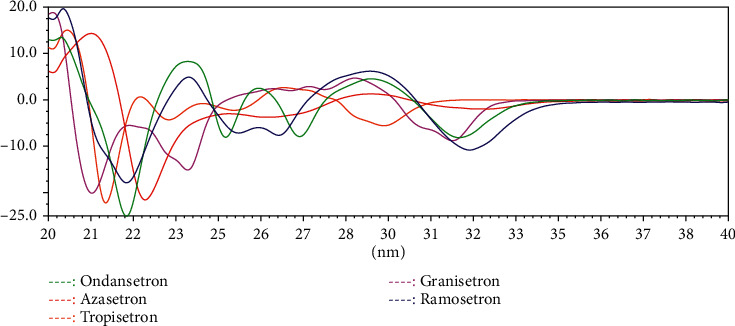
The 1st UV spectra of ODT, GNT, TPT, AZT, and RMT.

The differences in UV spectra are based on the differences in the structure of different 5-HT_3_ receptor antagonists (Figure 1). The results of the original and 1st UV spectral similarity are shown in Tables 3 and 4. According to the results, there are certain differences between the original UV spectra of the five 5-HT_3_ receptor antagonists. In particular, the 1st UV spectral similarity effectively magnified the difference in the original UV spectrum (Figures 4 and 5). Therefore, qualitative identification using 1st UV spectral similarity can effectively distinguish different 5-HT_3_ receptor antagonists.

The qualitative HPLC method is usually compared with the retention time of the reference substance. The retention time is influenced by several factors such as column packing, mobile phase, and instrument. It is difficult for the five 5-HT_3_ receptor antagonists with similar physical properties or structures to identify accurately using only the retention time. In this study, the relative retention time (RRT) was used to qualitatively determine the different 5-HT_3_ receptor antagonists. The reproducibility of the data could be improved by correcting the dead volume. In this experiment, the effects of different LC instruments (UltiMate 3000, Agilent 1260, and SHIMADZU LC-20A), brand columns (Agilent Zorbax Extend C_18_, InertSustain C_18_ column, and Kromasil C_18_), temperatures (±1°C), pH values (±0.2), and flow rates (±0.02) of the mobile phase on RRTs were investigated. The results are shown in Table 5. The results showed that the RRTs of the five 5-HT_3_ receptor antagonists were significantly different, but the changes in pH of the mobile phase and the chromatographic column had some effects on the RRTs. The differences between RRTs can mainly be attributed to different manufacturers being associated with the properties and preparation of packing materials. To this end, we herein restricted conditional parameters and performed spectral similarity as a double indicator to qualify the qualitative analysis.

### 3.4. Robustness Test of QAMS

To evaluate the robustness of the RCFs, the influence of different LC instruments (UltiMate 3000, Agilent 1260, and SHIMADZU LC-20A), brand columns (Agilent Zorbax Extend C_18_, InertSustain C_18_ column, and Kromasil C_18_), temperatures (±1°C), pH values (±0.2), and flow rates (±0.02) of the mobile phase on the RCFs was investigated. The RCFs of the five 5-HT_3_ receptor antagonists are shown in Table 6. The experimental results show that the values of RCF have good repeatability (RSDs ranging from 0.3% to 4.2%) under different experimental conditions. These results ensure that the HPLC-QAMS method can be well applied to routine analysis.

### 3.5. Sample Analysis

The developed HPLC-based QAMS method and ESM analytical method were applied to determine the five 5-HT_3_ receptor antagonists in their infusion samples and injection dosage form. The amounts of individual 5-HT_3_ receptor antagonists in injection dosage form and in infusion samples were calculated, and the results are listed in Table 7. From the comparative analysis results, we can conclude that there was no significant difference between the two analytical methods (using a *T*-test, *p* > 0.05), and the RSD values were <2.5%. Meanwhile, the contents of individual 5-HT_3_ receptor antagonists were also determined by HPLC methods described in the Chinese Pharmacopoeia (2015 Edition), and the results showed no significant difference between the above methods.

## 4. Conclusions

The developed HPLC-based QAMS and external standard analytical method are fast, selectively convenient, and sensitive to the simultaneous determination of ODT, GNT, TPT, AZT, and RMT in their injection dosage form and infusion samples. The comparative analysis results show no apparent distinction between the assay results of the two methods. The QAMS analysis method can provide reliable results, save reference materials, and shorten the analysis time. This method has great potential and can play an enhanced role in hospital-based quality control and quality assurance programs.

## Figures and Tables

**Table 1:** Regression equation, correlation coefficient (*r*), linear range, and detection limit of the five 5-HT3 receptor antagonists.

Compound	Regression equation	*r*	Linear range (*μ*g·mL^−1^)	Detection limit (*μ*g·mL^−1^)	Quantification limit (*μ*g·mL^−1^)
Azasetron	*Y* = 0.6321*X* + 1.3833	0.9996	5.0–100	0.2	0.6
Granisetron	*Y* = 0.6957*X* + 0.5444	0.9999	5.0–100	0.1	0.4
Tropisetron	*Y* = 1.0634*X* + 4.8592	0.9997	5.0–100	0.05	0.2
Ondansetron	*Y* = 1.8585*X* + 5.2914	0.9999	5.0–100	0.1	0.3
Ramosetron	*Y* = 2.3167*X* + 8.6551	0.9999	0.5–50	0.1	0.2

**Table 2:** Accuracy and precision results for the HPLC method.

Drug	Measured concentrations (*μ*g/mL)	Accuracy (%)	Precision RSD (%)	
			Intraday	Interday
Azasetron	10.0	99.7	1.5	1.7
	20.0	99.1	1.4	1.5
	50.0	99.2	1.2	1.8
Granisetron	10.0	101.7	0.7	1.1
	20.0	98.9	1.0	1.4
	50.0	99.0	0.8	1.4
Tropisetron	10.0	101.8	0.4	1.6
	20.0	99.3	1.6	1.8
	50.0	100.9	1.4	1.9
Ondansetron	10.0	101.8	1.1	1.5
	20.0	99.2	0.7	1.9
	50.0	98.3	0.9	1.7
Ramosetron	1.0	101.5	1.4	1.6
	3.0	101.6	1.3	1.6
	6.0	99.5	1.2	1.9

**Table 3:** UV spectra similarity of the five 5-HT3 receptor antagonists.

	Azasetron	Granisetron	Tropisetron	Ondansetron	Ramosetron
Azasetron	1.0000				
Granisetron	0.8964	1.0000			
Tropisetron	0.9049	0.9364	1.0000		
Ondansetron	0.8719	0.9166	0.9238	1.0000	
Ramosetron	0.8969	0.9164	0.9078	0.9823	1.0000

**Table 4:** 1st UV spectra similarity of the five 5-HT3 receptor antagonists.

	Azasetron	Granisetron	Tropisetron	Ondansetron	Ramosetron
Azasetron	1.0000				
Granisetron	0.1764	1.0000			
Tropisetron	0.1069	0.5472	1.0000		
Ondansetron	0.3419	0.3880	0.4861	1.0000	
Ramosetron	0.4371	0.5021	0.5515	0.8837	1.0000

**Table 5:** Effects of instrument, column, flow rate, pH value of mobile phase, and column temperature upon RRT of the five 5-HT_3_ receptor antagonists.

Compound	RRT	RSD (%)				
		Instrument	Column	Flow rate	Temperature	pH value
Azasetron	0.431	0.3	4.2	0.9	0.5	2.3
Granisetron	0.679	0.7	2.8	1.3	0.9	1.9
Tropisetron	0.812	0.3	2.1	0.4	1.2	2.5
Ondansetron	1.000	0	0	0	0	0
Ramosetron	1.132	0.5	2.6	0.6	0.6	1.6

**Table 6:** Effects of HPLC column, flow rate, pH value of mobile phase, and column temperature upon RCF of the five 5-HT_3_ receptor antagonists.

Compound	RCF	RSD (%)				
		Instrument	Column	Flow rate	Temperature	pH value
Azasetron	0.340	0.9	2.8	1.6	0.3	2.1
Granisetron	0.374	1.2	3.0	1.0	0.6	1.7
Tropisetron	0.572	0.8	2.3	1.5	1.2	2.2
Ondansetron	1.000	0	0	0	0	0
Ramosetron	1.247	1.5	4.2	1.3	0.8	2.5

**Table 7:** The results obtained by the QAMS and ESM methods (*n* = 3).

Compound	The QAMS methods		The external standard methods	
	Accuracy (%)	RSD (%)	Accuracy (%)	RSD (%)
*The five 5-HT*_*3*_ *receptor antagonists in injection dosage form*				
Azasetron	97.3	1.5	97.4	1.8
Granisetron	102.1	2.1	101.8	1.0
Tropisetron	101.7	0.9	102.1	1.6
Ondansetron	99.2	1.7	99.4	0.8
Ramosetron	101.3	2.4	100.9	1.4

*The five 5-HT*_*3*_ *receptor antagonists in infusion samples*				
Azasetron	94.9	1.3	95.1	1.5
Granisetron	100.3	0.8	99.8	0.7
Tropisetron	97.1	2.2	97.2	1.3
Ondansetron	103.6	1.9	103.8	1.2
Ramosetron	98.8	1.5	99.0	0.9

## Data Availability

The majority of the data used to support the findings of this study are included within the article. Other data are available from the corresponding author upon request.
